# Downregulation of *Klebsiella pneumoniae* RND efflux pump genes following indole signal produced by *Escherichia coli*

**DOI:** 10.1186/s12866-024-03443-w

**Published:** 2024-08-24

**Authors:** Galila G. Salama, Taghrid S. El-Mahdy, Walaa H. Moustafa, Mohamed Emara

**Affiliations:** 1 Faculty of Pharmacy, Department of Microbiology and Immunology, Helwan University, P.O. Box 11795, Ain-Helwan, Cairo, Egypt; 2 Department of Microbiology and Immunology, Faculty of Pharmacy, Modern University for Technology, and Information (MTI), Cairo, Egypt

**Keywords:** Signal molecules, Biofilm, Antibiotic resistance, Efflux pump

## Abstract

**Background:**

More than a century has passed since it was discovered that many bacteria produce indole, but research into the actual biological roles of this molecule is just now beginning. The influence of indole on bacterial virulence was extensively investigated in indole-producing bacteria like *Escherichia coli.* To gain a deeper comprehension of its functional role, this study investigated how indole at concentrations of 0.5-1.0 mM found in the supernatant of *Escherichia coli* stationary phase culture was able to alter the virulence of non-indole-producing bacteria, such as *Pseudomonas aeruginosa*, *Proteus mirabilis*, and *Klebsiella pneumoniae*, which are naturally exposed to indole in mixed infections with *Escherichia coli*.

**Results:**

Biofilm formation, antimicrobial susceptibility, and efflux pump activity were the three phenotypic tests that were assessed. Indole was found to influence antibiotic susceptibly of *Pseudomonas aeruginosa*, *Proteus mirabilis* and *Klebsiella pneumoniae* to ciprofloxacin, imipenem, ceftriaxone, ceftazidime, and amikacin through significant reduction in MIC with fold change ranged from 4 to 16. Biofilm production was partially abrogated in both 32/45 *Pseudomonas aeruginosa* and all eight *Proteus mirabilis*, while induced biofilm production was observed in 30/40 *Klebsiella pneumoniae*. Moreover, *acrAB* and *oqxAB*, which encode four genes responsible for resistance-nodulation-division multidrug efflux pumps in five isolates of *Klebsiella pneumoniae* were investigated genotypically using quantitative real-time (qRT)-PCR. This revealed that all four genes exhibited reduced expression indicated by 2^−ΔΔCT < 1 in indole-treated isolates compared to control group.

**Conclusion:**

The outcomes of qRT-PCR investigation of efflux pump expression have established a novel clear correlation of the molecular mechanism that lies beneath the influence of indole on bacterial antibiotic tolerance. This research provides novel perspectives on the various mechanisms and diverse biological functions of indole signaling and how it impacts the pathogenicity of non-indole-producing bacteria.

## Introduction

The discovery of antibiotics may be one of the greatest achievements in medicine, their introduction has transformed human and animal health systems by revolutionizing our weaponry in the war against infectious diseases. However, this health triumph was immediately ebbed by the subsequent realization that bacterial populations could quickly modify themselves to resist antimicrobials, propagate these resistance traits, and even share resistance genes with other contemporary bacteria within their environment. Such abilities have seriously compromised the usefulness of antibiotics in the war against microbes and warn of a future when antimicrobials may have very limited usefulness to control bacterial infection [1].

Therefore, many approaches were set to face the spread of antibiotic resistance, one approach that has received attention is the use of antibiotic adjuvants (resistance breakers and antibiotic potentiators). These are non-antibiotic compounds of natural or synthetic origin, that, when co-administered with antibiotics, act to block resistance or enhance antimicrobial activity. A potential candidate which meets the criteria is signal molecule indole [2].

The community of bacteria is intricate, natural environments of mutual support and competition are home to a wide variety of organism genera and species. So, for bacteria to survive and gain access to space, nutrients, and virulence, they must communicate through signaling molecules. Indole signaling has garnered growing attention as a novel class of signaling molecules, which, despite having many contradictions, have similar characteristics to quorum sensing molecules [3].

Several studies have shown that indole controls the formation of biofilms, antibiotic tolerance, virulence factors, plasmid stability, growth, and cell division in addition to being used as a biochemical test for bacterial identification [4]. The *tnaA* gene-encoded enzyme tryptophanase breaks down tryptophan into ammonia, pyruvate, and indole. *Escherichia coli* (*E. coli*) is one of more than 80 bacterial species known to produce indole but no pathway for indole degradation is reported for this bacterium [5]. Non-indole-producing bacteria may accept this signal molecule and cause alteration of their pathogenicity [6]. It is interesting to note that every bacterium reacts differently to exposure to indole.

The concentration of indole produced in the human gastrointestinal tract by the normal flora *E. coli* and its pathogenic opponent is found to be between 0.5 and 1 mM [7]. When this substantial amount of indole is introduced to non-indole-producing bacteria in mixed communities, they often use multiple monooxygenases or dioxygenases to produce derivatives of this signal molecule, which subsequently have the effect of changing their physiological function on several level [8].

The non-indole-producing bacteria in this study are part of a class of bacteria that resist different classes of antibiotics. They first appeared in hospitals and have been referred to as the ESKAPE (*Enterococcus faecium*, *Staphylococcus aureus*, *Klebsiella pneumoniae*, *Acinetobacter baumannii*, *Pseudomonas aeruginosa*, and *Enterobacter* spp.) group since 2008. Among those the Gram-negative bacilli namely, *Pseudomonas aeruginosa* (*P. aeruginosa*), *Proteus mirabilis (P. mirabilis)*, and *Klebsiella pneumoniae* (*K. pneumoniae*), were listed by the World Health Organization (WHO) as the most critical group of all due to their diverse resistance mechanisms which include enzymes that can destroy antibiotic and modify antibiotic bacterial targets [9]. As a result, an approach to find alternative anti-virulence tactics is gaining a recent interest [2].Based on our hypothesis, indole is believed to exert control over the expression of a wide array of genes and is involved in regulating the diverse physiological functions of non-indole-producing bacteria. The aim of this study is to gain insight into the effect of indole produced by *E. coli* on virulence and antibiotic tolerance of three medically important Gram–negative bacteria; *P. aeruginosa*, *P. mirabilis* and *K. pneumoniae*.

## Materials and methods

### Clinical isolates

A total of 93 isolates were recruited including 45 *P. aeruginosa*, 8 *P. mirabilis*, and 40 *K. pneumoniae*. Isolates were collected from Kasr El-Aini hospital over a period of months from November 2021 to July 2022. The clinical bacterial isolates were preidentified before their acquisition by VITEK 2 system, and subsequent verification was conducted by our side through biochemical tests prior to commencing any procedures. Stock culture of all bacterial isolates was then prepared and stored at − 80ºC in brain heart infusion (BHI) broth with 20% glycerol. The pathogenic strain of *E. coli* (ATCC 700728) was the indole-producing bacteria utilized in this work study. The strain was purchased from Faculty of Science, Helwan University.

### Preparation of indole from *E. coli* culture supernatant

A preculture medium was inoculated with a single colony of *E. coli* and incubated overnight at 37 °C. The culture was diluted 1:100 in fresh Luria Bertani broth (Sigma Aldrich, USA) supplemented with 1% tryptophan (Biolab, Hungary) and vortexed to get a uniform suspension. The uniform suspension of bacteria was incubated again overnight under dark conditions at 37 °C in shaking condition at 120 rpm [10].

### Preparation of crude indole extract

After incubation, culture supernatant (CS) was collected after centrifugation at 12,000 rpm for 15 min and was subjected to ultrasonic disintegration for 10 min. Supernatant was acidified by 1 N concentrated HCl (Chem-Lab, Belgium) to reach PH 2.5-3. To extract indole, double the ethyl acetate (LiChroslv, Germany) volume was added to acidified supernatant and shaken vigorously for 10 min in a separating funnel. The mixture was left at room temperature for 10 min to get the top layer of ethyl acetate. This layer is then used for further treatment.

In a rotary evaporator, the water bath temperature is set to 45 °C boiling temperature of ethyl acetate. The ethyl acetate layer was transferred to the round bottom flask and rotation was adjusted to avoid any bumping of the liquid sample. Upon complete evaporation of the liquid ethyl acetate, pure indole is left behind in the crystalline form attached to the bottom of the rotary flask. The crystals are re-dissolved in 20% methanol (PIOCHEM) and stored at -20 °C for future use [11].

### Qualitative and quantitative analysis of indole

The indole assay kit (Sigma Aldrich, USA) is based on a modified version of Ehlrich’s and Kovac’s reagents, which reacts with indole to produce a colored compound at 565 nm. The intensity of this colored compound is directly proportional to the indole in the sample. This kit is suitable for indole determination in biological samples (indole produced by indole positive bacteria) as followed through manufacturer’s recommendations. The indole was quantified in *E. coli* CS and as crude extract.

### Effect of indole on antibiotic tolerance

In this study, a total of five antibiotics, belonging to three different antibiotic classes, were utilized: ciprofloxacin (fluroquinolone), imipenem, ceftazidime, ceftriaxone, (Beta-lactams) and amikacin (aminoglycoside). The antibiotics were generously supplied by the Egyptian International Pharmaceutical Industries Company (EPICO).

The effect of indole on bacterial sensitivity to the antibiotics mentioned above was assessed by evaluating the alteration in the minimum inhibitory concentration (MIC) of antibiotics when combined with indole at concentrations varying from 0.07 to 0.5 mM and subsequent to the last preliminary optimization, a concentration of 0.15 mM was determined as the appropriate level to be employed for the ongoing investigation into antimicrobial susceptibility. MICs were determined using the microbroth dilution method, with each antibiotic tested at concentrations ranging from 256 to 0.0625 µg/µl, both in the absence and presence of 0.15 mM of indole crude extract added to each well following the guidelines set by the Clinical and Laboratory Standards Institute (CLSI), 2022. MIC values were recorded in absence and presence of indole with vehicle control done as well. The synergistic activity of this combination was reflected by reduction in MIC values compared to untreated isolates [12].

### Effect of indole on biofilm production

All isolates were screened for their ability to form biofilms by tissue culture plate method. Biofilm production was evaluated in tryticase soy broth (Difco, Sweden) with 1% glucose. Isolates from fresh agar plates were inoculated in respective media and incubated for 18 h at 37 °C in stationary condition and diluted 1:100 with fresh medium. Individual wells of 96 well-flat bottom plates were filled with 200 uL aliquots of the diluted cultures without and with indole at concentration of 0.2 mM added to each well, the indole concentration selected for continuation in the biofilm assay was determined following the conclusion of the last round of preliminary optimization. Vehicle control and negative control with only broth to check sterility, and non-specific binding were also carried out. The plates were then incubated for 16 h, 18 h and 24 h at 37 °C. After incubation, the content of each well was gently removed by tapping the plates. The wells were washed twice with 200 uL of phosphate buffer saline (PH 7.2) to remove free-floating planktonic bacteria [13].

For quantification of biofilms formed by adherent (sessile) organisms, the plate was dried at 50 °C for 30 min and the biofilm was then stained by addition of 1% crystal violet (CV). The liquid was discarded, and unbound CV was removed by washing with distilled water until transparent liquid was visually observed. The biofilm CV was solubilized in 33% glacial acetic acid and the absorbance was measured at 540–630 nm using absorbance microplate reader. Experiments were performed in triplicates and were independently repeated 2 times. The biofilm formation level of all isolates was categorized according to the classification system as non-adherent (OD ≤ ODC), weak (ODC < OD < 2ODC), moderate (2ODC < OD < 4ODC), and strong biofilm forming (4ODC < OD), where ODC (cut-off OD) is defined as three standard deviations above the mean OD of the negative control (blank value) [13].

### Efflux pump phenotypic detection

The phenotypic and qualitative detection of the efflux pump in *K. pneumoniae* isolates was performed by Cartwheel method. Briefly, the plates of tryptic soya agar culture media containing varying concentrations of ethidium bromide (EtBr) (1, 1.5, 2 and 2.5 mg/L) were prepared on the same day as the assay. Then the bacteria with 0.5 McFarland turbidity concentration were streaked on the plates. After 24 h of incubation at 37 °C, the plates were visualized under UV transilluminator and photographed. The isolates that had efflux pumps did not show emission of fluorescence. The capacity to efflux EtBr of each bacterial isolate was then ranked relative to the reference strain according to the following formula: Index = MC_EtBr_(K) - MC_EtBr_(REF) / MC_EtBr_(REF), where MC_EtBr_(K) and MC_EtBr_(REF) represent the minimum concentration of EtBr that produces fluorescence of the swabbed bacterial mass for the *K. pneumoniae* isolates and reference strain (*E. coli* ATCC 25922), respectively [14]. *K. pneumoniae* isolates which showed fluorescence index of ˃1.5 following a validation step using a higher EtBr concentration of 3 mg/l, were chosen for gene quantification analysis of resistance-nodulation-division (RND) efflux pumps.

### Quantification of the expression of the RND efflux pump genes (*acrA*, *acrB*, *oqxA and oqxB*) of Gram–negative bacteria using quantitative real-time (qRT)-PCR before and after indole exposure

Extraction of total bacterial RNA of five isolates of *K. pneumoniae* was done as previously described with minor modification. Briefly, a total of 100 µl of each bacterial suspension was added into the 96 well plates to determine MIC of antibiotics in absence and presence of indole according to CLSI guidelines as discussed above, then incubated for 18 h at 37 °C aerobically. After incubation, total RNA was isolated from bacterial suspension using a RNeasy mini kit (Qiagen, Germany) as per manufacturer’s instructions. The concentration and purity of the RNA was determined using a Nanodrop (Thermoscientific, USA) (260/280 ratio of > 1.8). The isolated RNA was reverse transcribed into cDNA using ReverAid RT Kit (ThermoFisher Scientific, USA) according to the manufacturer’s guidelines.

qRT-PCR was used to measure the relative expression of the RND-family efflux pump genes (*acrA*, *acrB*, *oqxA* and *oqxB*) using SYBR Green qPCR Master Mix (Xpert Fast SYBR (uni, Portugal) and the Bio-Rad platform in a total volume of 20 uL consisting of 2 uL cDNA, 2 uL 0.3–0.5 μm forward and reverse primers listed in Table 1, 10 uL master mix, and 6 uL nuclease free water. After a 3 min activation of the modified Taq polymerase at 95 °C, 40 cycles of 5s at 94 °C and 30 s at 60 °C were performed. In order to normalize the transcription levels of the target genes, the cycle threshold (CT) values of these genes were compared with the CT values of *rrsE* as a housekeeping gene, which was chosen as an endogenous reference. The expression of efflux pump genes in the tested isolates was then determined by calculating the relative expression of indole-treated isolates compared to non-treated isolates (which served as the control) in the presence of different antibiotics. Furthermore, the specificity of the generated products was verified through melting-point analysis. The 2^−ΔΔCT method was used to calculate the relative expression level of pump genes. A significant decrease of gene expression was concluded when the corresponding ratios were < 1.0. All reactions were performed in triplicate [15].

**Table 1:** Primers used for qRT-PCR

Target gene	Purpose	Primer	Sequence (5’-3’)	Ref
*rrsE*	Housekeeping gene 16 S rRNA	*rrsE*-F *rrsE*-R	CTACAATGGCATATACAA TTCTGATCTACGATTACT	[15]
*acrA*	RND efflux pump periplasmic part	*acrA*-F *acrA*-R	GGCAAACATGGATCAACTG GGCGGTATCGTAGTCTTG	
*acrB*	RND efflux pump transmembrane protein	*acrB*-F *acrB*-R	GGAAGATACACCGCAGTT TGTTAATGTCGCTGATGGA	
*oqxA*	RND efflux pump periplasmic part	*oqxA*-F *oqxA*-R	CGCAGCTTAACCTCGACTTCA ACACCGTCTTCTGCGAGACC	[16]
*oqxB*	RND efflux pump transmembrane protein	*oqxB*-F *oqxB*-R	ATCAGGCGCAGGTTCAGGT CGCCAGCTCATCCTTCACTT	

### Statistical analysis

The statistical analysis was performed using IBM SPSS Statistics software (version 22). For the comparison of means between two independent groups, the independent t-test and Mann-Whitney U test were used. The independent t-test was employed for analyzing qRT-PCR data to compare gene expression levels between groups. Additionally, Pearson correlation analysis was conducted to assess the relationship between variables of interest. All statistical tests were two-tailed, statistical significance was determined at *p* < 0.05.

## Results

### Indole production and quantification

Under the given experimental conditions, the CS obtained from the *E. coli* strain (ATCC 700728) produces indole contained an average of 200 ± 50 μm of indole. On the other hand, the crude extract had a quantified amount of 500 ± 65 μm indole and was utilized in all the subsequent experiments.

### Studying the impact of indole on antibiotic tolerance of non-indole-producing Gram-negative bacteria

**Table 2:** MIC fold change after exposure to indole crude extract at concentration of 0.15 mM

Gram-negative bacteria/total no. of isolates	Antibiotic	No of isolates experienced the following fold change					No. (%) of significant synergism
		1	2	4	8	16	
*P. aeruginosa* / 45	Ciprofloxacin	24	5	12	4	0	16 (35.6)
	Imipenem	19	2	15	5	4	24 (53.5)
	Ceftazidime	29	6	7	1	2	10 (22.2)
	Amikacin	26	5	8	6	0	14 (31.1)
*P. mirabilis* / 8	Ciprofloxacin	2	1	3	2	0	5 (62.5)
	Imipenem	2	1	3	2	0	5 (62.5)
	Ceftriaxone	2	2	3	1	0	4 (50)
	Amikacin	0	2	4	2	0	6 (75)
*K. pneumoniae* / 40	Ciprofloxacin	12	7	6	6	9	21 (52.5)
	Imipenem	3	4	8	13	12	33 (82.5)
	Ceftriaxone	27	2	2	5	4	11 (27.5)
	Amikacin	21	1	5	7	6	18 (45)

^a^(significant fold change is ≥ 4)

The determination of MIC was conducted for all isolates, with and without indole. The addition of indole exhibited a synergistic effect on all antibiotics as evidenced by a significant decrease *p* < 0.001in MIC values compared to the untreated isolates with fold change ranging from 4 to 16. The highest synergism was observed with imipenem while the lowest synergism was reported with third generation cephalosporins (ceftazidime and ceftriaxone) in all 3 Gram-negative bacteria tested *P. aeruginosa*, *P. mirabilis* and *K. pneumoniae* (Table 2).

### Studying the impact of indole on biofilm formation of non-indole-producing Gram-negative bacteria

**Table 3:** Fold change in biofilm after exposure to indole crude extract at concentration of 0.2 mM

Gram-negative bacteria	Fold change / no of isolates	Biofilm activity
*P. aeruginosa*	0.01/12 0.02/5 0.03/7 0.04/2 0.1/9	Reduced
*P. mirabilis*	0.01/6 0.02/1 0.1/1	Reduced
*K. pneumoniae*	-0.01/17 -0.02/6 -0.03/2 -0.04/2 -0.1/2	Induced

^a^Fold in –ve means induction of biofilm production

The biofilm forming ability was determined for all isolates, with and without indole. The addition of indole resulted in significant antibiofilm activity against all the eight *P. mirabilis* isolates and 32 of the 45 (71%) *P. aeruginosa* isolates. Contrary to these findings, 30 of the 40 (75%) *K. pneumoniae* isolates displayed a significant increase in biofilm activity after indole extract exposure *p* < 0.05 (Table 3).

### Efflux pump detection using Et-Br cartwheel method

**Fig. 1:**
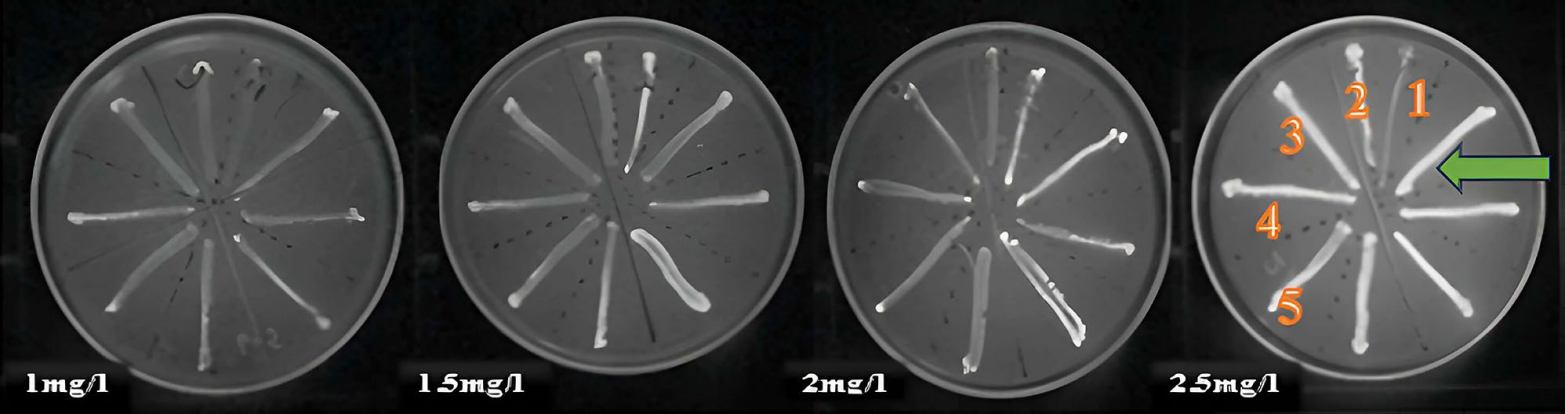
Et-Br cartwheel assay showing fluorescence of reference strain (*E. coli* ATCC 25922) marked with green arrow and eight *K. pneumoniae* isolates on agar plates containing increasing concentration of Et Br (1-2.5 mg/l). The five isolates with index > 1.5 are marked with numbers (1 to 5)

The method was implemented on the reference strain (*E. coli* ATCC 25922) and 21 *K. pneumoniae* isolates that were chosen based on their increased MIC fold change following indole exposure. To determine the efflux activity of the clinical isolates in comparison to the reference strain, their index for efflux activity was calculated as outlined in materials and methods. Out of 21 isolates, ten isolates had an index of 0.5, four isolates had an index of 1, and seven isolates had an index of 1.5. Five *K. pneumoniae* isolates of the seven achieving an index of ˃1.5 following validation step (as mentioned above) were chosen for gene quantification analysis of RND-type efflux as shown in Fig. 1.

### Expression analysis of efflux pump genes (*acrAB* and *oqxAB*)

**Fig. 2:**
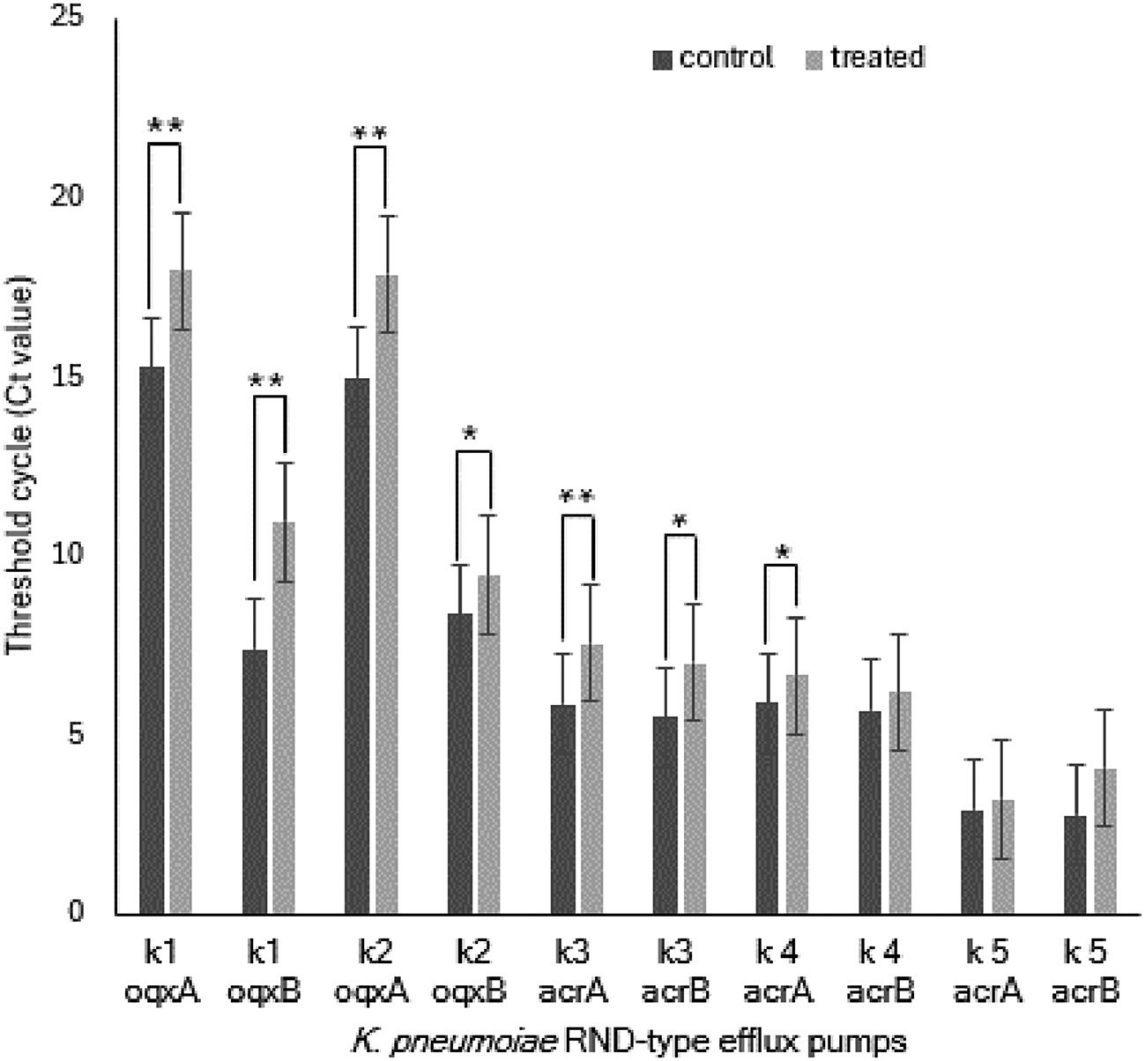
Fold changes in the expression of the *oqxA*, *oqxB*, *acrA,* and *acrB* genes in *K. pneumoniae* isolates. The bars represent relative expression levels of each indole-treated with corresponding control. Statistically significant differences are indicated by *p*-values of < 0.05 (*), and < 0.01 (**)

**Fig. 3:**
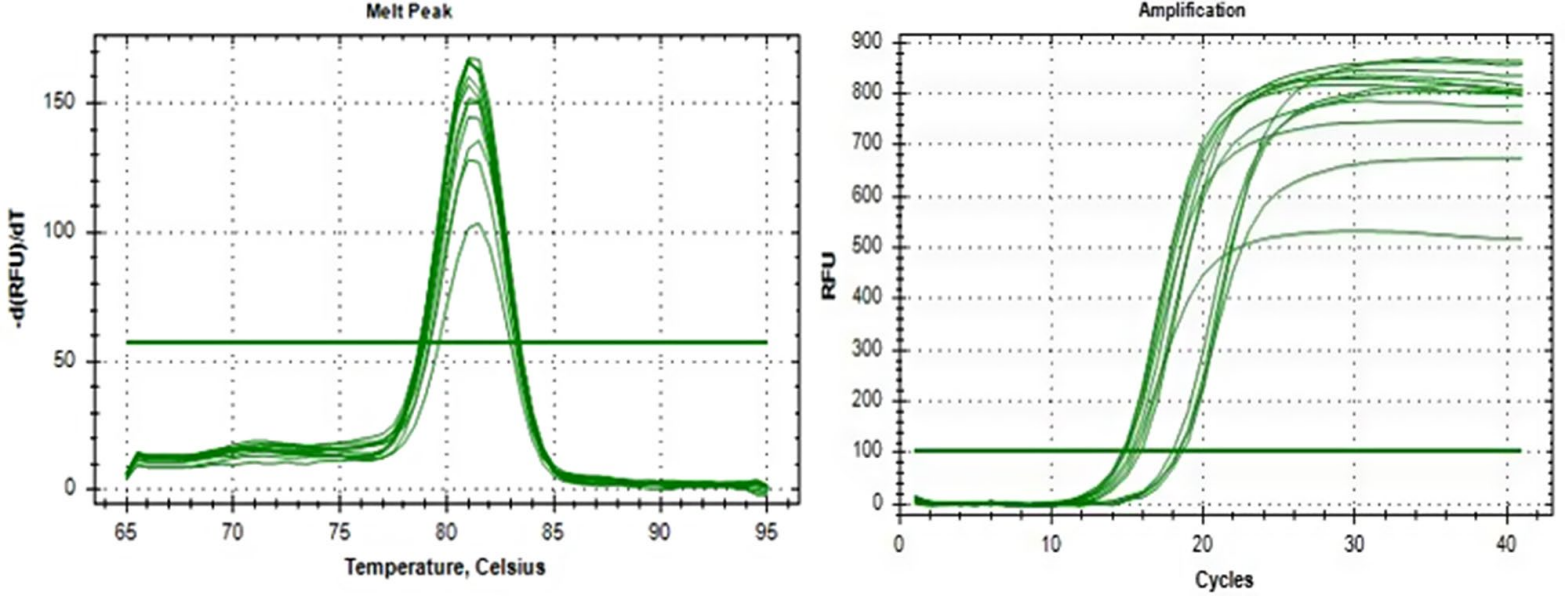
A representative experiment depicting dissociation curve on the left and amplification curve on the right, displaying the influence of indole on the expression of the RND-type efflux pump gene (*acrB*) in *K. pneumoniae* isolates

The expression of the *acrAB* and *oqxAB* efflux pump genes was examined in five isolates of *K. pneumoniae*. The results obtained from qRT-PCR indicated a significant downregulation *p* < 0.05 and *p* < 0.01of all four genes in the isolates treated with indole, compared to the control isolates shown in Table 4; Figs. 2 and 3.

**Table 4:** Downregulation of RND-type efflux in 5 *K. pneumoniae* isolates

Isolate	Examined antibiotic	Genes subunit A / 2^−ΔΔCT^	Genes subunit B / 2^−ΔΔCT^
1	Ciprofloxacin	oqx /0.153	oqx/0.086
2	Ciprofloxacin	oqx/0.139	oqx/0.46
3	Imipenem	acr/0.31	acr/0.351
4	Ceftriaxone	acr/0.483	acr/0.629
5	Amikacin	acr/0.799	acr/0.401

## Discussion

Many bacteria produce tiny, diffusible signal molecules like indole to coordinate multicellular actions such as virulence. It was found that exogenous indole accelerates the diffusion of antimicrobials across the cytoplasmic membrane, leading to hypothesis that indole influences the efficacy of antimicrobial compounds by increasing their propensity to diffuse across the bacterial cytoplasmic membrane where they could reach and build up their necessary lethal concentration [4, 8].

In the current study, the impact of indole on antibiotic tolerance was assayed with five antibiotics and it was proved that indole as a signal molecule had synergistic antibacterial effects with decreased MICs at 4–16 fold with most of tested isolates. Small molecules with conjugated aromatic rings, such as indole, are of high potential in efflux pump inhibitors research. These pumps are like intricate puzzle pieces, influencing bacterial physiology, metabolism, and pathogenicity [17]. The outcomes acquired through our research present clear and explanatory description to the underlying synergistic mechanism achieved by indole in combination with three different classes of antibiotic, and it underscores the capability of indole as a small signaling molecule to conquer bacterial resistance.

Formation of bacterial biofilm is initiated by cell surface adhesion and cell aggregation [18]. Scientists investigate thoroughly the mechanism whereby biofilms resist unfavorable environment using regulatory signal molecules. Among these mechanisms, the roles of indole appear to be increasingly prominent [19]. Biofilm data revealed a reduction in biofilm formation in *P. aeruginosa* and *P. mirabilis*, which we attributed to indole’s ability to reduce motility, chemotaxis, and cell attachment development in both indole and non-indole-producing bacteria. This finding is consistent with previous research that observed similar effects in *P. aeruginosa*, where the repression of the type IVb pilus flp–tad–rcp gene cluster and reduced swarming motility were observed [20]. Regarding *P. mirabilis*, it has been reported that the presence of indole leads to a decrease in the production of curli [11]. Similarly, in non-indole-producing bacteria such as *Acinetobacter oleivorans*, indole has been found to have a similar impact by reducing the folding of the QS regulator which, in turn, results in the differential expression of QS-controlled genes that are involved in the biofilm formation and motility regulation [21].During the investigation targeting indole-producing bacteria, particularly *Vibrio campbellii*, it was observed that the reduction in exopolysaccharide production, flagellar motility, and mRNA levels of the quorum sensing master regulator LuxR were all linked to the reduced biofilm effect of indole [22].

In the case of *k. pneumoniae* an induction of biofilm formation was detected which provides further evidence to support the theory that indole has the ability to stimulate the development of biofilm. One of proposed mechanisms is through the promotion of auto-aggregation, which enhances cell adhesion, surface conditioning, and subsequently facilitates the initiation of the biofilm by stimulating the synthesis of extracellular polymeric matrix [10]. A similar effect was also observed in indole-producing bacteria, specifically *Vibrio cholera*, where the genes responsible for the production of vibrio polysaccharide, a key component in biofilm formation, were found to be up-regulated in cells treated with indole [23].

It is worth noting that while indole consistently affects biofilm formation in various bacterial species, the specific direction of this effect (up-regulation or down-regulation) varies depending on the species. Additionally, indole has the capacity to selectively induce various oxygenases and can be further metabolized by bacteria, resulting in the production of numerous derivatives, these derivates were designed to behave as bacterial signals with indole or increase indole binding to the signal receptor [8]. This phenomenon elucidates the differential responses of individual pathogens to indole exposure, highlighting the variability in the functional role of specific signal molecules across different bacterial species.

The ultimate objective of this research was to analyze the impact of indole on genes associated with antibiotic resistance. Specifically, the focus was on efflux pump genes, which have recently garnered attention for their significant contribution to the multidrug resistance phenotype. Active efflux pumps have been demonstrated to have a significant impact on the development of antimicrobial resistance in bacteria. The MIC of the antibiotic substrate is typically 2–8 times higher in strains that overexpress efflux pump proteins compared to susceptible strains of the same species. Both antibiotic-susceptible and antibiotic-resistant bacteria possess efflux pump genes and proteins. These pumps can either be specific to a single substrate or can transport a variety of structurally dissimilar compounds, including antibiotics from multiple classes [24].

We focused our investigation on the genes responsible for producing the RND-type efflux pumps, AcrAB and OqxAB. These pumps consist of two main domains: a periplasmic part, AcrA and OqxA, and a transmembrane protein, AcrB and OqxB. However, the outer membrane protein is only present with the AcrAB subunit (known as ToIC) and absent with the OqxAB subunit. OqxAB is commonly found chromosomally in *K. pneumoniae* and usually plasmid located in other *Enterobacteriaceae* species. Their presence played a significant role in the emergence of decreased susceptibility and even resistance to olaquindox and other fluoroquinolone agents, including ciprofloxacin. Additionally, the substrate profile of AcrAB includes different classes of antibiotics, such as Beta-lactams, aminoglycosides, and tetracycline. The heightened virulence observed in *K. pneumoniae* in recent years can be attributed to the collective influence of overexpressed AcrAB and OqxAB [25].

Our approach involved analyzing the influence of indole on the expression of efflux pump genes in *K. pneumoniae*, specifically focusing on two distinct RND-type efflux pumps. Quantitative PCR analysis revealed a significant reduction in the expression levels of four target genes (*oqxA*, *oqxB*, *acrA*, and *acrB*) in indole-treated samples compared to the control.

A separate study also indicated that the MexGHI-OpmD efflux pump genes were significantly inhibited in *P. aeruginosa* following exposure to indole and 7-hydroxyindole [20]. Interestingly, contrary to earlier study, the introduction of indole actually stimulated the activity of the acrAB-tolC multidrug efflux system in *Salmonella enterica*, with this stimulation being regulated by the RamA regulator [26]. The novel findings in the current study regarding the reduced gene expression of RND-type efflux pump through indole signal molecule may have significant contribution to the existing knowledge in this field.

Moreover, one issue that may compromise the reproducibility of this research as well as data generalization is the small sample size may not be representative of the population.

## Conclusion

Each finding obtained in this study elucidates the distinct responses of pathogenic bacteria to indole as a signaling molecule. Moreover, this finding supports the notion of interconnection among different microorganisms through signaling molecules in a multi-species environment. According to our knowledge, this is the first report highlighting the capacity of indole to downregulate the expression of genes linked to efflux-mediated multidrug resistance in *K. pneumoniae*. Further investigations, both at the phenotypic and molecular levels, are imperative in order to unravel the more precise mechanism whereby indole and its derivatives regulate virulence. These studies would contribute to a better comprehension of indole signaling and aid in the identification of potential inhibitors for bacterial virulence.

## Data Availability

All data generated or analyzed during this study are included. The data set used and /or analyzed during the current study is available from the corresponding author on reasonable request.

## Abbreviations

*P. aeruginosa*: *Pseudomonas aeruginosa*
*P. mirabilis *: *Proteus mirabilis*
*K. pneumoniae*: *Klebsiella pneumoniae*
*E. coli*: *Escherichia coli*
RND: Resistance-nodulation-cell division
qRT-PCR: Quantitative real time polymerase chain reaction
CS: Culture supernatant
MIC: Minimum inhibitory concentration
CLSI: Clinical laboratory standard institute
CV: Crystal violet
OD: Optical density
Et-Br: Ethidium bromide
UV: Ultraviolet
RNA: Ribonucleic acid
flp: Fimbrial low-Molecular-Weight Protein
tad: Tight Adherence
rcp: RcpA Pilus Assembly Protein
QS: Quorum sensing
